# Molecular mechanisms of streptococcal disruption of the blood-brain barrier and their pathogenic role in bacterial meningitis

**DOI:** 10.3389/fimmu.2025.1628503

**Published:** 2026-01-07

**Authors:** Yumei Shi, Tao Liang, Chao Shi, Benhai Yao, Bo Xiong, Jun Zhang

**Affiliations:** 1 Department of Neurology, Affiliated Hospital of Zunyi Medical University, Zunyi, China; 2 The Neurological Disease Diagnosis and Treatment Center, Third People’s Hospital Affiliated to Zunyi Medical University, Zunyi, China

**Keywords:** bacterial meningitis, blood-brain barrier, molecular pathogenesis, neuroinflammation, streptococcal pathogens

## Abstract

Bacterial meningitis represents a devastating inflammatory disease of the central nervous system (CNS), characterized by the invasion of pathogens across the blood-brain barrier (BBB) and subsequent dysregulated immune responses. Key inflammatory mechanisms include pathogen recognition by microglial TLRs and NLRP3, neutrophil infiltration, and cytokine storms such as IL-1β and TNF-α, leading to BBB disruption, cerebral edema, and neuronal injury. Despite antimicrobial therapy, excessive inflammation often results in neurological sequelae. Emerging strategies target immunomodulation through inflammasome inhibitors and BBB preservation using nanoparticle drug delivery to mitigate inflammation-driven CNS damage. This review focuses on the intricate interplay between bacterial virulence factors and neuroinflammatory cascades, with particular emphasis on *Streptococcus pneumoniae* as a model pathogen. By integrating recent advances in molecular pathogenesis and translational immunology, this review provides a framework for developing precision therapies to mitigate inflammation-mediated CNS damage in bacterial meningitis.

## 1 Introduction

Bacterial meningitis is a severe CNS infection defined by purulent inflammation and pathogen detection in cerebrospinal fluid (CSF) (1, 2). *Streptococcus pneumoniae*, the leading cause, penetrates the blood–brain barrier (BBB) via hematogenous spread, triggering acute meningeal inflammation, with mortality rates up to 30% and long-term neurological sequelae in 50% of survivors (3). Additional pathogens, including Neisseria meningitidis, *group B Streptococcus*, and *Streptococcus suis*, contribute to its clinical heterogeneity (4, 5). Upon CNS entry, microbial toxins and an exacerbated host immune response elicit secondary brain injury, including ischemia, intracranial hypertension, and hydrocephalus (6). Concurrently, pathogen-derived toxins and dysregulated host immune responses exacerbate cerebral injury, culminating in pathological cascades such as cerebral ischemia, intracranial hypertension, and hydrocephalus (7). These processes ultimately result in irreversible neurological sequelae in over half of cured patients (8, 9).

Despite advancements in antimicrobial therapy, global morbidity and mortality remain unacceptably high, necessitating deeper mechanistic insights to overcome current therapeutic limitations (10). The dynamic molecular mechanisms underlying BBB disruption in pneumococcal meningitis and their correlation with neurological sequelae remain incompletely elucidated. Notably, the spatiotemporal regulation of pathogen-host interaction networks, particularly those involving signaling pathways governing BBB permeability, demands systematic exploration (11, 12). This review synthesizes current knowledge on the molecular mechanisms by which *Streptococcus* species compromise the BBB, with emphasis on the pathological cascades driving bacterial meningitis. By delineating these processes, we aim to provide a theoretical foundation for novel therapeutic strategies targeting BBB preservation, thereby improving clinical outcomes in patients afflicted by this devastating disease.

## 2 Acute pneumococcal meningitis and the BBB

### 2.1 Physiological structure and function of the BBB

The BBB represents a sophisticated neurovascular unit comprising specialized cerebral microvascular endothelial cells that interact bidirectionally with astrocytes, pericytes, and the extracellular matrix to establish and maintain central nervous system homeostasis through highly regulated tight junction complexes and coordinated intercellular signaling pathways (13, 14). Structurally, it consists of cerebral microvascular endothelial cells forming tight junctions, composed of transmembrane proteins and cytoplasmic anchors (zonula occludens), that restrict >99% of blood-borne pathogens while allowing regulated paracellular transport through junctional adhesion molecules (15, 16). The maintenance of BBB integrity depends on coordinated multicellular interactions where pericytes modulate angiogenesis through PDGFR-β signaling (17, 18), astrocytes secrete laminins to stabilize endothelial polarity (19, 20), and microglia maintain equilibrium between pro- and anti-inflammatory responses (21, 22). Physiological immune surveillance occurs through meningeal lymphatic drainage of cerebrospinal fluid components, including memory T cells (23, 24). Functionally, it operates through the physical exclusion of pathogens and inflammatory cells, alongside selective nutrient and waste transport, as well as immunomodulation by perivascular macrophages and endothelial pattern recognition receptors, which collectively prevent excessive inflammation (25, 26). In pneumococcal meningitis, pathogen virulence factors disrupt these mechanisms, collapsing CNS immune privilege and causing irreversible neurological damage.

### 2.2 Pneumococcal transmigration across the BBB into the CNS

Nasopharyngeal colonization by *Streptococcus pneumoniae* initiates pneumococcal meningitis pathogenesis (12, 27). Following mucosal breach, the pathogen spreads hematogenously through three main routes: middle ear invasion via the eustachian tube, alveolar dissemination, or direct vascular penetration causing bacteremia (28). Once in the bloodstream, pneumococcal virulence factors, including adhesins and cytolytic toxins (pneumolysin), disrupt the BBB by promoting endothelial cell adhesion, tight junction breakdown, and activation of inflammatory pathways. This cascade enhances BBB permeability, facilitating bacterial migration into the CNS (29). The interaction between bacterial effectors and host immune responses, alongside BBB reprogramming, forms the foundation for intracranial invasion (12, 29).

#### 2.2.1 Pneumococcal capsule in CNS invasion

*Streptococcus pneumoniae* employs the polysaccharide capsule as a key virulence factor for CNS invasion (30). The capsule’s dense structure sterically hinders complement deposition and phagocytic clearance, with encapsulated strains showing greater resistance to serum killing and neutrophil phagocytosis than unencapsulated variants (31). Paradoxically, while the capsule masks surface adhesins such as PspA/PspC, reducing mucosal colonization efficiency, *S. pneumoniae* dynamically regulates capsule expression via quorum sensing. During mucosal colonization, low-capsule phenotypes expose adhesins, whereas hematogenous dissemination triggers high-capsule expression for immune evasion (31–33). Pneumococcal surface proteins (Psps) orchestrate the pathogen’s triphasic invasion cascade spanning nasopharyngeal mucosa, bloodstream, and BBB through multimodal adhesion mechanisms. The pilus-associated adhesin RrgA mediates endothelial anchoring by specifically recognizing host polyimmunoglobulin receptor (plgR) and PECAM-1, establishing a molecular foothold for BBB breaching (34). Neuraminidase A synergistically enhances endothelial binding efficacy by targeting protein G-like lectin domains on the endothelium, with histopathological studies confirming that preferential adhesion of *S. pneumoniae* to subarachnoid vascular endothelia initiates CNS invasion, followed by dissemination along cortical and choroid plexus endothelial cells (35). The choline-binding protein (Cbp) family, which anchors to host cells via phosphorylcholine moieties, plays dual immunomodulatory and barrier-penetrating roles in pneumococcal pathogenesis. Specifically, PspC promotes endothelial adhesion through interaction with the polymeric immunoglobulin receptor (plgR), whereas CbpA facilitates transcytosis by engaging the platelet-activating factor receptor (PAFR) (29, 36). Concurrently, PLA2 acts as a critical inflammatory amplifier by hydrolyzing membrane phospholipids to generate arachidonic acid precursors. This process drives the aberrant upregulation of endothelial adhesion molecules, thereby exacerbating neuroinflammation. Notably, elevated plasma PLA2 levels exhibit a positive correlation with disease severity in pneumococcal meningitis, supporting its utility as a biomarker for monitoring neuroinflammatory progression (37).

#### 2.2.2 Dual mechanisms of pneumolysin in BBB disruption and neuroinflammation

Pneumolysin (Ply) is a cholesterol-dependent, pore-forming toxin that contributes to BBB disruption through both direct cytotoxicity and immunomodulatory effects (38, 39). It forms transmembrane pores approximately 19–30 nm in diameter via its β-barrel domain, resulting in the collapse of ion gradients and subsequent lytic cell death (40). Mechanistically, Ply exerts direct cytotoxic effects by disrupting (41, 42) in cerebral microvascular endothelial cells, inducing retraction of astrocytic end-feet, and triggering pyroptosis in microglia, all of which collectively compromise BBB integrity (43). In parallel, Ply modulates host inflammatory signaling by dose-dependently activating the NLRP3 inflammasome, thereby promoting the maturation of IL-1β and IL-18. It also stimulates the TLR4/NF-κB signaling axis, leading to increased production of chemokines such as CXCL8 and CCL2, which facilitate neutrophil transendothelial migration and amplify neuroinflammatory cascades (44). Notably, Ply concentrations activate membrane repair mechanisms, fostering an immunosuppressive microenvironment through IL-10 secretion and TLR2 signaling inhibition, enabling bacterial immune evasion. This dysregulation of pro-/anti-inflammatory equilibrium underpins tissue damage and pathogen dissemination in pneumococcal meningitis (45, 46). Furthermore, Ply exacerbates CNS microenvironmental imbalance by impairing meningeal ciliary clearance, collapsing neuronal mitochondrial membrane potential, and inducing astrocytic cytoskeletal disorganization, collectively driving irreversible neurological deficits (47–49).

### 2.3 Role of host inflammatory responses in determining outcomes of pneumococcal meningitis

#### 2.3.1 Brain microvascular endothelial cells

Brain microvascular endothelial cells (BMECs) form the structural core of the BBB, providing a frontline defense against hematogenous pathogens such as *Streptococcus pneumoniae* and *S. suis*. These cells establish high transendothelial resistance via tight (occludin, claudin-5, ZO-1) and adherens (VE-cadherin) junctions (50). During streptococcal meningitis, BMECs undergo pathogenic reprogramming in response to microbial adhesins, pore-forming toxins, and systemic inflammation (51). Through pattern recognition receptors (TLR2, TLR4, NOD-like receptors), BMECs detect pathogen-associated molecular patterns (PAMPs) like lipoteichoic acid, pneumolysin, and suilysin. Activation of TLR2/4–MyD88 signaling upregulates proinflammatory cytokines (IL-1β, TNF-α), chemokines (CXCL8, CCL2), and adhesion molecules (ICAM-1, VCAM-1), facilitating leukocyte recruitment (52, 53). In addition, streptococcal virulence factors exert direct cytotoxic effects on BMECs. Pneumolysin disrupts ion gradients and triggers necroptosis or pyroptosis via β-barrel pore formation; suilysin induces Ca²^+^-dependent calpain activation, destabilizing cytoskeletal integrity (51). Histological analyses reveal endothelial swelling, junctional disassembly, and actin collapse, enhancing BBB permeability (3). BMECs also shape CNS immunity by secreting IL-6, TNF-α, and CXCL10, promoting Th1 polarization and microglial activation. Moreover, NLRP3 inflammasome activation in BMECs—via toxin-induced K^+^ efflux and mitochondrial stress—leads to IL-1β/IL-18 maturation and pyroptosis (54). Thus, BMECs function not only as structural barriers but as immunologic sentinels central to CNS invasion and neuroinflammation.

#### 2.3.2 Microglia

The pathogenesis of streptococcal meningitis involves a complex interplay between microbial virulence factors and coordinated responses from the neurovascular unit components. Microglia, as the CNS’s primary immunoregulatory cells, initiate the inflammatory cascade through pattern recognition receptors such as TLR2, TLR4, andNLPR3. These receptors specifically detect pneumococcal pathogen-associated molecular patterns including pneumolysin, lipoteichoic acid, and peptidoglycan fragments (55). This recognition triggers MyD88/NF-κB-dependent cytokine production, including IL-1β, TNF-α and CXCL1, which facilitates neutrophil recruitment and adaptive immune activation via MHC-II and co-stimulatory molecules (CD80/86). Microglia further contribute to host defense through phagolysosomal degradation and antimicrobial peptide secretion, particularly cathelicidin LL-37 (55). However, excessive activation leads to neurotoxic effects including glutamate excitotoxicity and reactive oxygen species overproduction. Concurrently, astrocytes and pericytes modulate the inflammatory milieu and blood-brain barrier integrity. Astrocytes regulate ion/water homeostasis through aquaporin-4 channels while secreting pro-inflammatory cytokines, specifically interleukin-6 and CCL2, following Toll-like receptor activation (56, 57). Pericytes respond to bacterial toxins by altering PDGFR-β/TGF-β signaling pathways, resulting in basement membrane remodeling and increased endothelial permeability (58). This integrated neurovascular response demonstrates a delicate balance between protective immunity and pathological neuroinflammation, where microglial activation states, astrocytic responses, and pericyte-mediated vascular changes collectively determine disease progression and clinical outcomes in pneumococcal meningitis (55, 58, 59).

#### 2.3.3 Spatiotemporal regulation of neuroinflammation in pneumococcal meningitis

During pneumococcal meningitis, pattern recognition receptors (PRRs) initiate spatially and temporally regulated immune cascades. TLR2 recognizes bacterial peptidoglycan/lipoteichoic acid, TLR4-MD2 detects the β-sheet domain of pneumolysin, and endosomal TLR9 senses CpG-rich DNA via MyD88-dependent signaling, contingent on pathogen internalization (60–62). These PRRs activate NF-κB/MAPK pathways, triggering IL-6, TNF-α, and CXCL8 release, while simultaneously assembling the NLRP3 inflammasome complex (NLRP3, ASC, caspase-1), which cleaves pro-IL-1β and pro-IL-18 into active cytokines. IL-1β propagates neuroinflammation via positive feedback, and IL-18 enhances IFN-γ via STAT1–IRF1 signaling, exacerbating Th1-driven immunopathology and oxidative stress (63). PRR hyperactivation also induces release of mitochondrial DAMPs (mtDNA), fueling a self-amplifying cycle of tissue damage and secondary immune activation that promotes BBB breakdown and neuronal injury (60, 63). PRR-mediated microglial activation triggers a neurovascular cascade, where endothelial IL-6/TNF-α signaling via JNK/AP-1 and astrocytic IL-1β via NF-κB drive pro-inflammatory microenvironments (64, 65). CXCL12/CXCR4 arrests leukocytes at the BBB, while CCL2/CCR2 and CXCL1/CXCR2 facilitate monocyte/neutrophil transmigration (66). Neutrophils contribute to bacterial clearance through NETosis and α-defensin secretion but also inflict tissue damage via ROS and elastase-mediated ECM degradation (67, 68). Knockout models underscore dual roles: neutrophil depletion attenuates inflammation yet elevates bacterial burden, revealing compartment-specific functions (68). Infiltrating leukocytes amplify inflammation via IL-1β/caspase-1 pyroptosis, TNF-α/NF-κB-induced CAM expression, and IFN-γ/STAT1/IRF1-mediated CXCL10 production, driving Th1 polarization (69–71). Cytokine networks show nonlinearity. For instance, IL-6 knockout reduces edema but increases mortality, while IL-1R deficiency weakens the BBB yet enhances bacterial clearance, emphasizing the need for spatiotemporally balanced immunomodulation in therapy (72, 73).

#### 2.3.4 Reactive oxygen and nitrogen species

Reactive oxygen/nitrogen species (RONS) drive neuroinflammation in pneumococcal meningitis through three axes: pathogen-driven *S. pneumoniae* autolysis releases H_2_O_2_, which reacts with NO· to form peroxynitrite (ONOO^-^), inducing lipid peroxidation and mitochondrial DNA damage; enzymatic neutrophil myeloperoxidase (MPO) generates HOCl from H_2_O_2_/Cl^-^, activating MMP-9 to degrade collagen IV and disrupt BBB integrity; and metabolic IFN-γ-induced NOS2 sustains NO·, which reacts with O_2_^-^ to form ONOO^-^, nitrating occludin/ZO-1 and destabilizing tight junctions. CSF nitrate/nitrite levels correlate with BBB damage severity (74–76). RONS exhibit duality: endothelial NOS1 (eNOS) maintains microvascular integrity via PI3K/Akt, while NOS2 knockout reduces edema and cytokines, underscoring spatiotemporal specificity (77). Combined catalase/SOD therapy reduces CSF leukocytes by 50% and suppresses caspase-3-dependent apoptosis, supporting RONS-targeted strategies (75–77).

## 3 Streptococcal breach of the blood-brain barrier

Streptococcal pathogens invade the BBB via surface adhesins binding endothelial receptors PECAM-1 and laminin receptor, degrading tight junction proteins occludin and claudin-5, and activating MMP-2/9 to hydrolyze collagen IV, enabling transendothelial migration (78). Post-invasion, PAMPs activate NLRP3 inflammasomes via TLR2/4-NF-κB, driving caspase-1-dependent IL-1β/IL-18 release and generating reactive species (O_2_^-^, ONOO^-^), perpetuating a pathogen-inflammation-injury cycle (79). *S. pneumoniae* upregulates NLRP3/ASC via STAT6-SPDEF during nasopharyngeal colonization, while sialylated serotype-specific capsules enhance CNS penetration (80–82). *S. suis* serotype 2 secretes suilysin to induce endothelial pyroptosis and modulates BBB-penetrating genes (*srtA*, *fbps*) via VirR/VirS two-component signaling (83, 84). *S. equi subsp. zooepidemicus* and *group B Streptococcus* exploit adhesins and surface proteins for transcytosis, highlighting conserved yet diverse neuroinvasive strategies.

### 3.1 Disruption of tight junction proteins

The BBB tight junctions (TJs), composed of ZO-1/2, claudin-5, occludin, and JAMs, maintain CNS homeostasis via a high-resistance paracellular barrier (85). *Streptococci* disrupt TJs through oxidative stress, *Streptococcus pneumoniae* H_2_O_2_ is converted by myeloperoxidase (MPO) to HOCl, activating MMP-9 to degrade occludin and claudin-5, enzymatic cleavage, *S. suis* Stk kinase ubiquitinates claudin-5 via SMURF1, enhancing proteasomal degradation, and epigenetic regulation, *group B Streptococcus* suppresses TJ transcription via NF-κB/Snail1, redistributing ZO-1 and claudin-5 (74, 75, 86–91). *S. pneumoniae* infection elevates CSF MMP-8/9, while MMP inhibitors reduce BBB permeability (86, 87). *Stk* knockout (Δ*stk*) reduces microvascular adhesion by 80% and impairs RhoA/ROCK-driven cytoskeletal contraction, confirming its dual role in TJ disruption (88, 89). Therapeutic strategies including claudin-5-stabilizing peptides and ROCK inhibitors restore BBB resistance, highlighting their translational potential (90, 91).

### 3.2 Activation of immune responses

**Figure 1:**
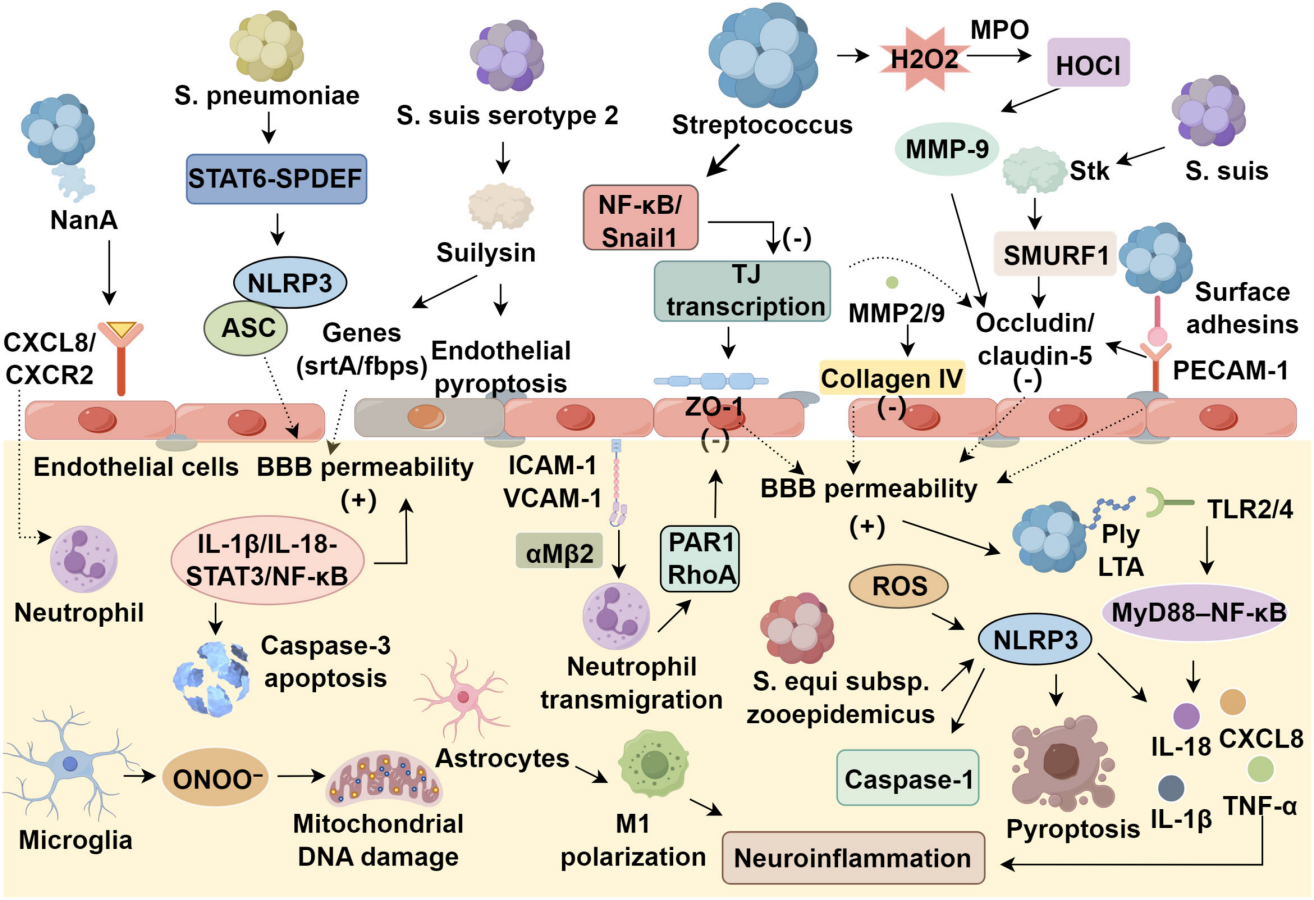
Molecular mechanisms of streptococcal disruption of the blood-brain barrier.

The CNS innate immune system responds to streptococcal invasion of the BBB through the recognition of lipoteichoic acid (LTA) and pneumolysin (Ply) by TLR2/4 recognition of LTA/Ply, activating MyD88/NF-κB to drive IL-1β/TNF-α/CXCL8 crosstalk. In parallel, activation of the NLRP3 inflammasome complex comprising ASC and caspase-1 is triggered by potassium efflux and reactive oxygen species, resulting in IL-18 secretion and pyroptotic cell death (43). Additionally, endothelial expression of adhesion molecules ICAM-1 and VCAM-1 is upregulated, promoting neutrophil transmigration via integrin αMβ2; the extent of leukocyte infiltration positively correlates with CSF levels of matrix metalloproteinase-9 (MMP-9) (92). However, immune hyperactivation exacerbates injury: neutrophil elastase disrupts ZO-1 via PAR1/RhoA; microglial ONOO^-^ induces mitochondrial DNA damage; and IL-1β/IL-18-STAT3/NF-κB crosstalk triples BBB permeability, triggering hippocampal caspase-3 apoptosis (92). Astrocytes worsen neuroinflammation via glutamate-glutamine excitotoxicity and macrophage M1 polarization, highlighting the need for balanced immune modulation (92). *Streptococcus pneumoniae* induces CNS inflammation through TLR2-MyD88-dependent microglial NF-κB activation by peptidoglycan/teichoic acid, and C1q-MBL-mediated complement activation by capsular polysaccharides, forming MACs that lyse neurons (60, 93–95). These processes elevate IL-6, TNF-α, and IL-1β in endothelia/glia. While caspase-1 knockout preserves BBB integrity by reducing IL-1β, IL-1R deficiency exacerbates leakage via compensatory TLR4-TRIF signaling (69, 70, 96–98). *S. pneumoniae* further recruit neutrophils through NanA-induced CXCL8/CXCR2 activation, correlating with CSF bacterial load (99). Other *Streptococcus suis* induces IL-1β via MyD88/TLR2-AIM2, while small RNA *rss04* stabilizes TLR4 by inhibiting TRIM32 ubiquitination, reducing occludin phosphorylation (100–103). In *S. equi* subsp. *zooepidemicus*, triggers NLRP3/caspase-1 via K^+^ efflux, with miR-223-3p suppressing NLRP3-dependent IL-18 (104). Chemokine remodeling during pneumococcal infection drives neutrophil (CXCR1/2) and monocyte (CCR2/5) recruitment (105). NLRP6 knockout paradoxically improves survival despite increasing NETs, implicating gasdermin D in regulation (106, 107). Critical for therapeutic development, IL-1β maturation is controlled by both AIM2/NLRP3 inflammasomes and neutrophil proteases (108, 109), highlighting key targets to modulate neuroinflammation in streptococcal infections (**Figure 1**).

### 3.3 Synergistic invasion strategies of cytolytic toxins and metabolic enzymes

Streptococcal pathogens breach the BBB via membrane-targeting virulence factors: *Streptococcus suis* suilysin (Sly) forms 30–50 nm pores in endothelial membranes, inducing Ca²^+^-dependent calpain activation and an increase in phospholipase A2 (PLA2G3) activity, which hydrolyzes lipids to disrupt barrier integrity. Wild-type *sly* strains exhibit higher BBB penetration than mutants (84, 110). *Streptococcus pneumoniae* pneumolysin triggers p38 MAPK/NFATc1 signaling via pre-pore complexes, upregulating IL-1α and reducing endothelial resistance. Sublytic Ply inhibits autophagy via PI3K/Akt/mTOR, inducing mitochondrial collapse and caspase-9-dependent apoptosis (51, 111, 112). *S. suis* enolase (Eno) binds ribosomal protein RPSA, activating p38/ERK-eIF4E to elevate HSPD1 and induce cytoskeletal rearrangements, forming intercellular gaps. Eno also stimulates TLR4/MyD88-dependent IL-8 secretion, exacerbating edema and enhancing BBB penetration (113–115). These findings reveal *Streptococcus* employs synergistic toxin-enzyme strategies, offering novel therapeutic targets (51, 110–115) (**Table 1**).

**Table 1:** Molecular mechanisms of streptococcal BBB disruption.

Virulence factor	Mechanism of action	Pathophysiological effect	Associated pathogens
Surface adhesins (CbpA, MRP)	Bind endothelial receptors (PECAM-1, laminin receptor), disrupt tight junctions (claudin-5, occludin)	Paracellular barrier degradation; enhanced bacterial transmigration	*S. pneumoniae*, *S. suis*
Pneumolysin/Suilysin	Cholesterol-dependent pore formation; NLRP3 inflammasome activation via TLR4/NF-κB	Endothelial pyroptosis, oxidative stress (RONS), IL-1β/IL-18	*S. pneumoniae*, *S. suis*
Capsular polysaccharides	Immune evasion via complement inhibition (C3b/iC3b); sialylation enhances CNS tropism	Reduced phagocytosis; serotype-specific BBB penetration (19F, 6B)	*S. pneumoniae*, *group B Streptococcus*
Host-derived MMPs	MMP-9 activation via ROS/cytokines (IL-1β, TNF-α)	Collagen IV degradation; TJ protein (ZO-1, occludin) disruption	All streptococcal pathogens
RONS (O_2_^-^, ONOO^-^)	Generated by neutrophil MPO, microglial NOS2; nitrates TJ proteins	Mitochondrial DNA damage; BBB hyperpermeability (↑ caspase-3 apoptosis)	*S. pneumoniae*, *S. suis*, *group B Streptococcus*
Neuraminidase A (NanA)	Cleaves endothelial sialic acid residues; activates CXCL8/CXCR2	Neutrophil infiltration (r = 0.76 with CSF bacterial load)	*S. pneumoniae*

## 4 Molecular mechanisms of immune dysregulation and neural injury

Following *Streptococcus pneumoniae* breach of the BBB, capsular polysaccharides mediate immune evasion via C3b/iC3b complement inhibition and resisting neutrophil extracellular trap (NET) adhesion, while simultaneously activating microglial TLR4/MyD88 signaling to drive excessive IL-6, IL-1β, and TNF-α, establishing a pro-inflammatory cascade (116–118). This response precipitates oxidative stress and cytokine storms, leading to mitochondrial DNA damage and caspase-3–dependent neuronal apoptosis in the hippocampus—pathologies strongly correlated with long-term cognitive deficits in meningitis survivors (119, 120). Notably, N-acetylcysteine (NAC) reduces BBB permeability and restores cognitive function scores by inhibiting NF-κB nuclear translocation, whereas doxycycline improve long-term neurological outcomes by preventing MMP-9-mediated ZO-1 degradation (120).

*Streptococcus pneumoniae* exhibits spatiotemporal virulence heterogeneity: high-pneumolysin (Ply) strains activate ATG5/LC3-II–mediated autophagy and enable bacterial clearance via p62/SQSTM1, whereas low-Ply strains inhibit autophagosome maturation and show 2.3-fold higher BBB penetration (121, 122). Similarly, *Streptococcus suis* Sly forms 25–30 nm pores to activate NLRP3 inflammasomes, increasing IL-18 secretion fivefold, an effect reversible by cryptotanshinone through Sly oligomerization blockade (123, 124). Additionally, *S. suis* also upregulates ATG7/Beclin1 via IFN-γ/STAT1, inducing aberrant autophagy in brain endothelia and reducing occludin phosphorylation (125). *Group B Streptococcus* employs complementary evasion strategies, including sialylated capsule-mediated complement resistance and PilA-triggered CXCL8 secretion via α2β1 integrin-FAK signaling, which enhances neutrophil transmigration (126–129). The CovR/CiaR regulatory system further modulates invasion by suppressing capsular gene *cpsE*, tripling BBB penetration in serotype III strains, though CovR phosphorylation inhibitors restore barrier integrity (127, 128). Streptococci thus subvert BBB defense through C3 convertase inhibition, autophagy suppression, and immunosuppressive IL-10/TGF-β upregulation. To overcome therapeutic delivery barriers, LDL receptor–targeting lipoprotein-mimetic β-lactamase inhibitors offer a promising nanodelivery strategy for CNS infections (117, 118, 121, 122).

## 5 Conclusion

The molecular interplay between streptococcal virulence factors and BBB defenses underscores the complexity of bacterial meningitis pathogenesis. Key mechanisms, ranging from adhesion mediated tight junction degradation and toxin driven endothelial pyroptosis to reactive oxygen and nitrogen species cytokine crosstalk, highlight the pathogen’s ability to exploit host inflammatory responses for central nervous system invasion. Paradoxically, excessive immune activation exacerbates neural injury, emphasizing the need for balanced immunomodulation. While current therapies remain limited by BBB impermeability and antibiotic resistance, advances in nanotechnology such as LDLR targeted drug delivery and virulence factor inhibitors like cryptotanshinone against SLY present novel avenues for intervention. Future research must prioritize spatiotemporal mapping of pathogen host interactions, particularly the roles of microglial polarization and meningeal lymphatic drainage in disease progression. A multidisciplinary approach integrating pathogen specific strategies and BBB preservation is essential to reduce the global burden of meningitis related morbidity and mortality.
